# Plant Antimicrobials for Food Quality and Safety: Recent Views and Future Challenges

**DOI:** 10.3390/foods12122315

**Published:** 2023-06-08

**Authors:** Loris Pinto, Melvin R. Tapia-Rodríguez, Federico Baruzzi, Jesús Fernando Ayala-Zavala

**Affiliations:** 1 Institute of Sciences of Food Production, National Research Council of Italy, Via G. Amendola 122/O, 70126 Bari, Italy; federico.baruzzi@ispa.cnr.it; 2 Departamento de Biotecnología y Ciencias Alimentarias, Instituto Tecnológico de Sonora, 5 de Febrero 818 sur, Col. Centro, Ciudad Obregón, Obregón 85000, Sonora, Mexico; melvin.tapia14987@potros.itson.edu.mx; 3 Centro de Investigación en Alimentación y Desarrollo, A.C, Carretera Gustavo Enrique Astiazarán Rosas 46, Hermosillo 83304, Sonora, Mexico; jayala@ciad.mx

**Keywords:** antimicrobial activity, essential oils, food preservation, foodborne pathogens, polyphenols

## Abstract

The increasing demand for natural, safe, and sustainable food preservation methods drove research towards the use of plant antimicrobials as an alternative to synthetic preservatives. This review article comprehensively discussed the potential applications of plant extracts, essential oils, and their compounds as antimicrobial agents in the food industry. The antimicrobial properties of several plant-derived substances against foodborne pathogens and spoilage microorganisms, along with their modes of action, factors affecting their efficacy, and potential negative sensory impacts, were presented. The review highlighted the synergistic or additive effects displayed by combinations of plant antimicrobials, as well as the successful integration of plant extracts with food technologies ensuring an improved hurdle effect, which can enhance food safety and shelf life. The review likewise emphasized the need for further research in fields such as mode of action, optimized formulations, sensory properties, safety assessment, regulatory aspects, eco-friendly production methods, and consumer education. By addressing these gaps, plant antimicrobials can pave the way for more effective, safe, and sustainable food preservation strategies in the future.

## 1. Introduction

The use of natural antimicrobials in the food industry is gaining attention due to the consumers’ demand for environmentally friendly production systems and products with clean labels, promoting the use of natural antimicrobial preservatives rather than synthetic ones [1,2]. Indeed, synthetic food preservatives such as nitrates, benzoates, sulfites, sorbates, and formaldehyde are known for allergic or carcinogenic effects [3]. Microbial food spoilage is responsible for about 25% of food losses [4]. According to the Food and Agriculture Organization (FAO), wasted food costs approximately 680$ billion in industrialized countries and 310$ billion in developing countries, with a high emission footprint for meat products [5]. Moreover, the growing consumption of fresh, minimally processed, and ready-to-eat foods increases the chance of microbial contamination by spoilage and pathogenic microorganisms [1]. Therefore, natural antimicrobials should be promoted to extend the shelf life of perishable foods, and to ensure the product’s microbial food safety.

Plant antimicrobials represent the main group of natural preservatives, including secondary metabolites targeting microbial cells. Different parts of plants, such as seeds, fruit, peels, leaves, and roots are rich in plant antimicrobials such as phenolic compounds (e.g., simple phenols, phenolic acids, anthocyanins, flavonoids, quinones), tannins, essential oils and terpenoids, glucosinolates derivatives, alkaloids, and thiols [6,7]. Most of the plant extracts are generally recognized as safe (GRAS) and were granted the qualified presumption of safety (QPS) status in the USA and EU, respectively [8]. Plant extracts, such as moso bamboo (Takeguard™) with benzoquinone derivatives and tannin, or an antifungal blend (Biovia™ YM10) with green tea (*Camellia sinensis* L.) extract and mustard (*Brassica nigra* W.D.J. Koch) essential oil, are commercially available as alternatives to chemical preservatives [1]. Moreover, the European Food Safety Authority (EFSA) authorized rosemary (*Rosmarinus officinalis* L.) extract, endowed with antimicrobial activity, as a food additive (E 392) [9,10].

Plant antimicrobials were proposed to control the growth of microbial spoilage populations and foodborne pathogens. As regards the control of spoilage microorganisms, several applications were described in animal-based foods. In fish products, grape (*Vitis vinifera* L.) seed extract, tea polyphenols, thyme essential oil, and rosemary extract delayed the growth of lactic acid bacteria, *Enterobacteriaceae*, hydrogen sulfide-producing bacteria (HSPB), and psychrotrophic bacteria, well known to produce off-flavours [11]. In meat products, tannic acid or catechin showed good antimicrobial activity in camel sausages, whereas ethanolic extracts of rosemary and clove (*Syzygium aromaticum* L.) reduced spoilage bacterial counts in raw chicken meat. In beef sausages, the use of *Ziziphus* leaf extracts, rich in vanillic and ellagic acids, inhibited the growth of spoilage bacteria during cold storage [12]. Among essential oils, the application of *Ziziphora clinopodioides* Lam., rich in carvacrol, thymol, p-cymene, and γ-terpinene, showed the best antimicrobial activity against spoilage bacteria in beef patties [13]. In plant-based foods, as reviewed by Patrignani et al. [14], citral, hexanal, and 2-(E)-hexenal showed antimicrobial activity against yeasts responsible for spoilage of fresh-cut fruits, soft drinks, and fruit-based salads, whereas citral-based films or the application of oregano (*Origanum vulgare* L.) and thyme (*Thymus vulgaris* L.) oil during the washing step reduced spoilage bacterial populations on salad. The antimicrobial action of plant extracts against foodborne pathogens is well documented [1,2,3,7,15,16,17]. In particular, phenolic extracts and essential oils showed remarkable antibacterial action against Gram-positive and Gram-negative bacteria, including spore-forming bacteria. In addition to the effect against viable cells, plant antimicrobials inhibited the production of microbial toxins [18,19] and biofilm formation [20,21,22].

Despite the antimicrobial action of plant antimicrobials, their use in the food industry is hampered by chemical instability, limited dispersibility in food matrices, limited availability of ready-to-use commercial formulations, or unacceptable flavour profiles [6]. For these reasons, several stabilization techniques, such as nano-emulsions, encapsulation, and inclusion in active packaging, were proposed [6,23,24]. Moreover, these stabilization techniques ensure, in some cases, better antimicrobial activity of the bioactive compounds, and a controlled release during food storage.

However, some challenges remain, including potential negative sensory impacts, variations in antimicrobial effectiveness, and concerns about the possible development of microbial resistance. To address these issues, researchers explored synergistic combinations of plant antimicrobials and the application of hurdle technologies, which involve the simultaneous or successive use of multiple preservation techniques. Although plant extracts showed considerable potential in food preservation, limited information is available concerning their safety. In some instances, these extracts can be contaminated with various hazardous substances, such as heavy metals [25], mycotoxins [26], or crop protection residues [27]. The levels of contamination in plant extracts are affected by several factors, including the cultivation practices employed, the geographical location of the cultivation site, and the application of crop protection products. Further research is needed to establish proper guidelines and regulatory frameworks that can help minimize the risks associated with contaminants in plant extracts, ultimately ensuring the safe application of these natural preservatives in the food industry. Further research is also necessary to understand the modes of action of plant antimicrobials alone or in combination to optimize their formulation and the delivery of bioactive compounds. Addressing these gaps will help the acceptance of plant extracts as food preservatives and their use in different food industries.

This review aims to summarize the applications of plant antimicrobials in the food sector. After that, an overview of different classes of plant antimicrobials, antimicrobial activity against spoilage, and pathogenic microorganisms in different foods is described. Then, the stabilization techniques of plant extracts are presented followed by their use in different food matrices. This review also discusses the additive and synergistic effects of various combinations of plant antimicrobials, as well as the integration of plant extracts into different hurdle technologies, including mild or non-thermal treatments, to enhance food preservation. Finally, safety aspects and regulation related to the use of plant extracts are introduced. By providing a comprehensive overview of the current knowledge, this review aims to contribute to the ongoing development and optimization of food preservation techniques based on plant antimicrobials. 

## 2. Classification and Antimicrobial Activity of Plant Antimicrobials

A great diversity of structures among plant secondary metabolites (PSMs) occurs in nature (e.g., more than 12,000 known alkaloids, more than 10,000 phenolic compounds, and over 25,000 different terpenoids) [1]. From a structural point of view, plant antimicrobials can be divided in two classes: PSMs with one or several nitrogen atoms into their structures, such as alkaloids, glucosinolates, and PSMs without nitrogen, such as terpenoids and phenolic substances. Alkaloids, glucosinolates, and phenolic substances are water-soluble compounds, whereas terpenoids are lipophilic PSMs [28]. The following sections summarize the different classes of plant antimicrobials and their antimicrobial action against main food-related microorganisms.

### 2.1. Polyphenols

Polyphenols are PSMs produced by higher plants, sharing a common chemical structure characterized by at least one aromatic ring with one or more hydroxyl groups [29]. Polyphenols can be classified as flavonoids and nonflavonoids. The latter includes the phenolic acids (e.g., derivatives of benzoic acid and cinnamic acid), stilbenes (e.g., resveratrol), tannins (e.g., proanthocyanidins, gallotannins, and ellagitannins), and lignins (e.g., secoisolariciresinol). Flavonoids can be divided into six subclasses: flavonols, flavones, flavanones, flavanols, anthocyanins, and isoflavones [30].

#### 2.1.1. Phenolic Acids

Phenolic acids are divided into hydroxybenzoic acids (e.g., vanillic, gallic, salicylic, syringic, and protocatechuic acid) and hydroxycinnamic acids (ferulic, rosmarinic, p-coumaric, chlorogenic, cinnamic, and caffeic acid) [31]. The main phenolic acids showing antimicrobial action are gallic acid, ferulic acid, and p-coumaric acid [31]. A minimum inhibitory concentration (MIC) of 1000–2000 µg mL^−1^ was found for gallic acid and ferulic acid against *Escherichia coli*, *Staphylococcus aureus*, and *Listeria monocytogenes* [32]. Ferulic acid and p-coumaric acid showed MIC values of 500–1000 µg mL^−1^ against *Salmonella enteritidis* [33]. Li et al. [34] recently found that p-coumaric acid controlled the contamination of *Alicyclobacillus acidoterrestris* in apple juice. However, the antibacterial action of phenolic acids can be enhanced considering their derivatives, as demonstrated for alkyl ferulate and gallate esters against *L. monocytogenes* and *E. coli*, respectively [35,36]. Regarding the antifungal action, ferulic acid and p-coumaric acid showed antifungal activity against *Botrytis cinerea* and *Alternaria alternata* [37,38]. As reported for the antibacterial action, ester derivatives of phenolic acids showed enhanced antifungal action compared to phenolic acids. In particular, ethyl p-coumarate showed interesting antifungal activity against *Alt. alternata* [39].

#### 2.1.2. Stilbenes, Tannins, and Lignins

Other polyphenols endowed with antimicrobial activity are stilbenoids, tannins, and lignins. Stilbenes such as resveratrol showed antibacterial action against foodborne pathogens, with MIC values of 100–200 µg mL^−1^ for *S. aureus* and *Enterococcus faecalis*, and >200 µg mL^−1^ for *E. coli* and *Sal. enterica* [40]. Cai et al. [41] found that pterostilbene had higher antifungal activity against ochratoxin A (OTA)-producing *Aspergillus carbonarius* than piceatannol and resveratrol. As regards tannins, they are classified into hydrolysable and condensed tannins. Hydrolysable tannins such as ellagitannins showed antibacterial action against *S. aureus* and *E. coli*. In particular, increased free galloyl groups enhanced antibacterial action against *S. aureus*, while large molecular size positively affected the antimicrobial effect against *E. coli* [42]. Condensed tannins such as proanthocyanidins from persimmon [43] or chokeberry [44] showed MIC values of 0.7–5 mg mL^−1^ against *S. aureus*. Regarding the antibacterial activity of lignin, different sources and extraction processes can result in different antibacterial performances. However, the ethanol fractionation of bamboo kraft lignin enhanced the antibacterial activity compared to non-fractionated lignin, and the ethanol fraction showed a MIC value of 2 mg mL^−1^ against *Bacillus subtilis* and *S. aureus* [45]. 

#### 2.1.3. Flavonoids

Flavonoids are the main dietary polyphenols. They show a characteristic phenyl-benzopyrone structure and can be classified into anthocyanidins, flavan-3-ols, flavones, flavanones, flavonols, and isoflavonoids [29]. Among them, flavan-3-ols, flavonols, and flavanones showed the highest antibacterial activity against foodborne pathogens [31]. In particular, flavan-3-ols such as epigallocatechin-3-gallate showed antibiofilm activity against *L. monocytogenes* [46], and bactericidal effect against *E. coli* [47]. The main flavonol endowed with antibacterial activity is resveratrol. Resveratrol showed a MIC value lower than 10 mg mL^−1^ against *E. coli* O157:H7 and *Sal. enteritidis* [48] and 300–600 μg mL^−1^ against methicillin-resistant *S. aureus* [49]. However, the presence of rhamnose and additional hydroxyl groups in the flavonoids myricetin-3-O-rhamnoside and quercetin-3-O-rhamnoside resulted in reduced antibacterial activity compared to quercetin [31]. As regards the antifungal activity of flavonoids, quercetin at 0.25 mg mL^−1^ inhibited mycelial growth of *Penicillium expansum* [50] and showed a MIC value of 505 μg mL^−1^ against *Aspergillus flavus* [51]. Flavanones belong to a sub-class of flavonoids. The most interesting antibacterial activity was found for sophoraflavanone G against methicillin-resistant *S. aureus*, with MIC values ranging from 0.5 to 8 µg mL^−1^ [52]. Recently, other flavonoids, such as the mono-prenylated isoflavonoids showed high antifungal activity against *Zygosaccharomyces parabailii*, a spoilage yeast of acidic food products, with a minimum fungicidal concentration (MFC) of 12.5 μg mL^−1^ [53].

### 2.2. Terpenes and Essential Oils

Essential oils (EOs) are complex blends of aromatic metabolites extracted from different plant parts, including leaves, bark, flowers, and roots, using solvents, distillation, or microwaves [54]. Volatile compounds represent 90–95% of EOs, including monoterpenes, sesquiterpene hydrocarbons and their oxygenated derivatives, aldehydes, alcohols, and esters. The non-volatile portion (5–10% of the whole EO) comprises hydrocarbons, fatty acids, sterols, carotenoids, waxes, cumarines, and flavonoids. The main antimicrobial compounds present in EOs can be divided into different groups: terpenes (e.g., p-cymene, limonene), terpenoids (e.g., thymol, carvacrol), and phenylpropenes (e.g., eugenol, vanillin) [30]. 

Rosemary EO, rich in the monoterpenes α-pinene, 1,8-cineol, and camphor, showed antibacterial action against *E. coli* and *S. aureus* [55,56]. A recent study [57] showed that the geographic origin of rosemary EOs affected their composition and antimicrobial activity. EOs extracted from *Salvia officinalis* L., *Lavandula dentata* L., and *Laurus nobilis* L., rich in 1,8-cineol, inhibited the growth rate of *A. carbonarius* and the OTA production [58]. 

EOs with terpenoids such as thymol and carvacrol as main compounds paid great attention due to their broad spectrum of antimicrobial activity and potential application through direct contact and vapour phase. Oregano and thyme EOs showed antibacterial activity by direct contact against drug-resistant Gram-positive pathogens such as *S. aureus* and *Enterococcus faecium*, and Gram-negative pathogens such as *E. coli* and *Sal. thyphimurium* [59,60]. These EOs showed antimicrobial activity in vapour phase, with MIC values of 0.16–4.00 μg mL^−1^ of air against *E. coli* and *Penicillium expansum* [61]. Moreover, oregano and thyme EOs vapours showed antifungal activity against different species of the genera *Aspergillus*, with MIC values of 15.6–62.5 μL L^−1^ of air [62]. Regarding p-cymene, this monoterpene has low antibacterial activity, high MIC values, and no antifungal action against *Rhizopus oryzae* and *A. niger* [63]. Similarly, in *B. cinerea*, *P. italicum*, and *Alt. alternata*, p-cymene showed higher MIC values than other monoterpenes such as thymol and γ-terpinene [64].

Phenylpropanoids such as eugenol and isoeugenol, both present in clove EO, showed antibacterial action against *E. coli* and *L. monocytogenes* with MIC values in the range 312.5–625 µg mL^−1^ [65]. Clove oil, with eugenol as the main compound, inhibited *P. italicum* growth on citrus fruit when applied at concentrations ranging from 0.05% to 0.8% (*v*/*v*) [66]. Other phenylpropanoids, such as vanillin, showed a bacteriostatic effect against foodborne pathogens, but MIC values were higher than that of pure compounds belonging to terpenes or terpenoids [67].

Other bioactive compounds occurring in EOs are the aldehydes citral and cinnamaldehyde, found in lemongrass (*Cymbopogon citratus* Stapf) EO and cinnamon (*Cinnamomum verum* Presl) bark EO, respectively. Free citral showed a MIC value of 0.8 mg mL^−1^ against *B. cereus* and 2 mg mL^−1^ against *E. coli* and *S. aureus* [68]. However, the main application of citral is its use as an antifungal agent, as demonstrated against different fungal strains [69,70,71]. As regards cinnamaldehyde, it showed higher antibacterial activity than cinnamon oil against Gram-positive bacteria [72]. Cinnamaldehyde at 150 μg mL^−1^ inhibited the spore production and mycelial growth of *A. niger* [73] and showed antifungal activity and alternariol reduction at 0.200 μL mL^−1^ against *Alt. alternata* [74].

### 2.3. Glucosinolate Derivatives

Glucosinolates are the main bioactive compounds of *Brassica* plants. The breakdown of glucosinolates releases nitriles, thiocyanates, and isothiocyanates. In particular, isothiocyanates, largely occurring in cruciferous vegetables, are the most reactive compounds endowed with antimicrobial activity. Allyl-, benzyl-, and 4-methylsulfinylbutyl isothiocyanates are the main compounds with antimicrobial activity against bacterial pathogens and fungi [7]. Allyl-isothiocyanate at the concentration of 1 µL L^−1^ reduced of 4 log cfu g^−1^ the *Sal. thyphimurium* load on lettuce [75], whereas at 0.1% *v*/*w* inhibited *L. monocytogenes* growth in chickpea puree stored for 10 days at 4 °C [76]. Allyl-isothiocyanate showed antifungal activity against *A. flavus* in maize and *P. verrucosum* in barley, reducing the aflatoxin B1 and ochratoxin A accumulation, respectively [77,78]. Benzyl-isothiocyanate showed MIC values ranging from 60 to 160 µM against enterotoxigenic *E. coli* [79], and 120 µM against *L. monocytogenes* [80]. Benzyl-isothiocyanate at 25 µg mL^−1^ inhibited the growth of *A. carbonarius* and *A. ochraceus*, whereas *A. niger* was more resistant to both allyl- and benzyl-isothiocyanates than other aspergilli [81]. Other bioactive isothiocyanates are sulforaphane (4-methylsulfinylbutyl isothiocyanate) and phenethyl isothiocyanate. Both compounds showed MIC values of 40–88 mg mL^−1^ against *S. aureus* and *E. coli* [82]. However, their use for applications in the food sector is limited compared to allyl- and benzyl-isothiocyanates. Other isothiocyanates demonstrated an interesting antifungal activity. In particular, the volatile compound 2-phenylethyl isothiocyanate showed a MIC value of 1.2 mM against *Alt. alternata*, and reduced the development of the black spot rot on pear [83], whereas 2-(4-methoxyphenyl)ethyl isothiocyanate showed an EC_50_ value of 4.2 μg mL^−1^ against *A. niger*, and inhibited the spore germination by 95% [84].

### 2.4. Alkaloids and Thiols

Several plant extracts include alkaloids and thiols as antimicrobial compounds. Alkaloids are PSMs classified based on their chemical structure and natural origin. Although more than 18.000 alkaloids are known, mainly represented by plant alkaloids [85], their use in the food sector is limited due to their well-known toxic and neuroactive effects. Recently, berberine, an isoquinoline alkaloid found in roots and stem-bark of *Berberis* plants, was the most studied alkaloid exploited for its antimicrobial activity against food-related microorganisms and was proposed as a food preservative [86,87,88]. In particular, *Berberis vulgaris* root and leaf extracts, rich in berberine, showed a MIC value of 150 µg mL^−1^ against *E. coli* and *S. aureus*, and 60–100 µg mL^−1^ against different *Aspergillus* species [86]. Berberine at 1.6 mg mL^−1^ inhibited mycelial growth and spore germination of *P. italicum* [88].

As regards thiols, the main antimicrobial compounds are allicin and its derivatives [29]. Allicin is a sulphur compound occurring in garlic, effective against spoilage yeasts, Gram-positive and Gram-negative foodborne pathogens, with MIC values lower than 30 µg mL^−1^ [89]. The main oxidation derivatives of allicin are diallyl disulphide and diallyl trisulfide. Diallyl disulphide showed antibacterial action against *B. cereus* and a MIC value of 120 µg mL^−1^ [90], whereas diallyl trisulfide treatment reduced, by 1.5 log cfu g^−1^, the *Campylobacter jejuni* count on chicken [91].

### 2.5. Modes of Action

**Figure 1:**
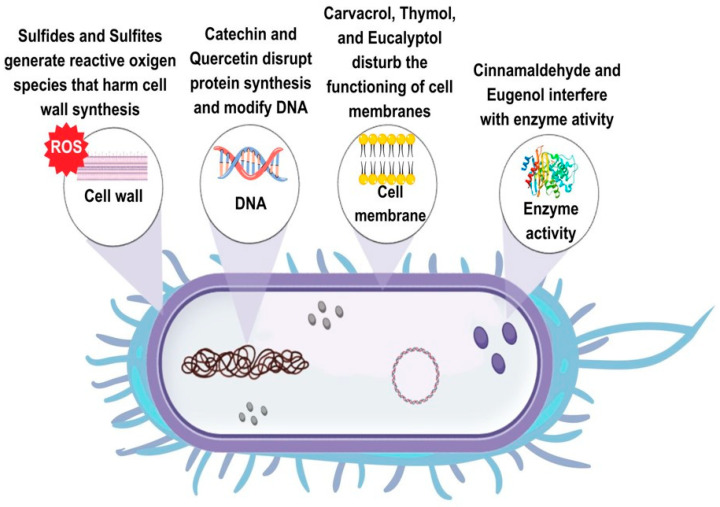
Mechanisms of action of plant antimicrobials against foodborne bacteria.

PSMs described in the previous sections have multiple mechanisms of antimicrobial action (Figure 1). In particular, different cell targets are affected by exposure to polyphenolic substances, essential oil compounds, isothiocyanates, alkaloids, and thiols. As regards polyphenols, the three main mechanisms of action are the modification of the membrane permeability, the intracellular enzyme inactivation, and the modification of fungal morphology. Additional mechanisms of antimicrobial action of polyphenols are the modification of intracellular pH, the interference with the ATP-generating system, and the inhibition of DNA synthesis [1]. 

Different polyphenolic classes have specific mechanisms of action. Phenolic acids mainly interact with the cell membrane intercalating the phospholipid layer, or crossing the membrane, decreasing the intracellular pH, and/or interacting with cellular constituents [92]. The antibacterial action of phenolic acids against *L. monocytogenes* depends on their dissociated/undissociated form. In particular, chlorogenic acid and gallic acid reduced extracellular pH, caffeic acid, p-hydroxybenzoic acid, protocatechuic acid, and vanillic acid were active in their undissociated form, and p-coumaric acid and ferulic acid showed antibacterial action in both dissociated and undissociated form [92]. In *Sal. enteritidis*, chlorogenic acid treatment damaged intracellular and outer membranes and inactivated key enzymes of the tricarboxylic acid cycle (TCA) [93]. Phenolic acid esters showed multiple mechanisms of antibacterial action, such as the damage of bacterial membranes, changes in the conformation of protein membranes, formation of complexes with bacterial DNA, and oxidative damage [35,36]. As regards the antifungal mechanism of phenolic acids, it is well known that these compounds produce oxidative stress and disorganization of the wall or membrane of the hyphae [94], but, as in the case of *B. cinerea*, they can also affect the ATP synthesis and cellular metabolism acting as an uncoupler of oxidative phosphorylation [95]. Resveratrol inhibits ATP synthesis, hydrolysis, and cell division in *E. coli* [40]. Pterostilbene treatment induces incomplete sporangia, membrane rupture, and downregulation of the biosynthetic genes of the OTA production in *A. carbonarius* [41]. The disruption of cell membranes and functions is the primary mode of antibacterial action of tannins. However, the inhibition of microbial enzymes, the deprivation of the nutrients required for the microbial growth, and the inhibition of oxidative phosphorylation were also suggested [42]. In *P. digitatum*, tannins disrupted the cell wall and caused the leakage of intracellular content [96]. The antibacterial modes of action of lignin are the damage of the cell membrane through its phenolic compounds, the decrease in intracellular pH, and the increase in osmotic pressure [97]. Flavonoids have multiple modes of antimicrobial action. Quercetin inhibited DNA gyrase, increased membrane permeability, and prevented ATP synthesis in *E. coli* [29], whereas in *S. aureus*, it inhibited key enzymes necessary for the protein synthesis [31]. Flavonols such as quercetin, rutin, morin, rhamnetin, and flavones such as acacetin and apigenin have membrane-disrupting activity. Conversely, flavanones naringenin and sophoraflavanone G reduce the fluidity in regions of both inner and outer cellular membranes [18]. Catechins such as epigallocatechin gallate, at high concentration, generated reactive oxygen species (ROS), causing membrane damage [18]. As regards the antifungal mechanism of flavonoids, a recent study showed that quercetin downregulated genes involved in the conidial and mycelial development, while reducing the production of aflatoxin probably by lowering levels of ROS [51]. Flavonoids from the medicinal–edible plant *Sedum aizoon* L. damaged the cell membrane and the cell wall, and interfered with the mitochondrial respiratory metabolism, the protein biosynthesis, and the amino acid metabolism in *P. italicum* [98].

As regards the antibacterial mechanism of action of terpenoids and essential oils, these compounds can disrupt cell walls and cytoplasmic membranes, increasing their permeability. Essential oils can also solidify the cytoplasm, damage lipids and proteins in the cell, and inhibit bacterial enzymes [29]. Specifically, terpenoids such as carvacrol disrupted the cell membranes and inhibited the respiratory activity in *L. monocytogenes* [99], while it increased the cell permeability and reduced the ATP levels in *E. coli* [100]. In *Sal. enteritidis*, the antibacterial action of oregano essential oil was mainly attributed to thymol rather than its isomer carvacrol, with changes in the protein regulation and the DNA synthesis [101]. The antibacterial action of terpenes such as limonene was associated with increased cell permeability, inhibition of the ATP synthesis, dysfunction of the respiratory chain complex, and inhibition of the transcription of nucleic acids [102,103,104]. Phenylpropenes such as eugenol altered the membrane permeability in *E. coli* [105], whereas they increased reactive oxygen species, depolarized the membrane potential, and decreased the ATP content in *Shigella flexneri* [106].

The antifungal mechanisms of action of essential oil compounds such as thymol and carvacrol are related to changes in the morphology of hyphae, the increase in membrane permeability, and the reduction in total lipids and ergosterol content [107,108]. As regards p-cymene, the antibacterial mode of action is related to the expansion of the cytoplasmic membrane and a moderate generation of ROS [63]. Other essential oil compounds, such as citral and geraniol, showed distinctively antifungal mechanisms of action. In particular, citral downregulated the sporulation- and growth-related genes in *A. flavus* and *A. ochraceus*, whereas geraniol determined intracellular ROS accumulation in *A. flavus* and increased cell membrane permeability in *A. ochraceus* [70]. Oxidative stress was partially responsible for the antifungal action of cinnamaldehyde against *A. niger*, causing cell damage and increasing membrane permeability [73]. Citral, limonene, and eugenol damaged the cell membranes and destroyed the yeast proteins in *Zygosaccharomyces rouxii* [109]. In *A. carbonarius*, eugenol determined the leakage of cytoplasmic contents, increased the lipid peroxidation, decreased the ergosterol content, increased the membrane permeability, and induced oxidative stress [110].

Luciano and Holley [111] demonstrated that allyl isothiocyanate inhibited thioredoxin reductase and acetate kinase in *E. coli* O157:H7. The bacteriostatic/fungistatic effects of benzyl isothiocyanate against *E. coli*, *B. subtilis*, *Sal. enterica*, *S. aureus*, *A. niger*, and *P. citrinum* were associated with interferences with the ATP production, enzymes and coenzymes of the energy metabolism [112]. Conversely, in *B. cinerea*, benzyl isothiocyanate disrupted the plasma membrane integrity and induced ROS accumulation in the spores, inhibiting their germination [113]. Other glucosinolate derivatives, such as sulforaphane and phenethyl isothiocyanate, are effective against different pathogenic bacteria by inhibiting the synthesis of nucleic acids or disrupting the membrane integrity depending on bacterial species [83]. 

The alkaloid berberine binds to the FtsZ protein, causing the inhibition of bacterial cell division [114]. In *Sal. typhimurium*, it reduces the number of type I fimbriae and prevents biofilm formation [115]. In fungi, berberine damages the plasma membrane integrity and reduces the contents of soluble proteins and reducing sugars. In addition, a high H_2_O_2_ content was found in berberine-treated *P. italicum* mycelia [88]. Thiols such as allicin display antimicrobial action due to the rapid reaction of thiosulfinates with thiol groups of key enzymes [89]. 

## 3. Plant Antimicrobials for Food Quality and Safety

In the past, the use of plant material during traditional food processing was defined empirically to improve the sensory characteristics of the food and the food safety and quality levels. It should be considered that several spices, obtained from different plant species, often include antimicrobial molecules and are usually supplemented to foods as flavouring agents. For this reason, the use of plant compounds as food preservatives is close to traditional recipes and, therefore, highly accepted by consumers. This section presents the direct application of plant antimicrobials in different foods, highlighting the antimicrobial action against spoilage and pathogenic microorganisms.

### 3.1. Plant Antimicrobials as Food Preservatives

Plant antimicrobials were exploited as preservatives in several foods to control the microbial growth of food spoilage microorganisms or foodborne pathogens [116]. This section summarizes recent published results, focusing on the direct application of plant extracts or their bioactive compounds as preservatives in food products (Table 1). 

#### 3.1.1. Applications in Plant Foods

This section presents the applications of whole plant extracts or their antimicrobial compounds against spoilage microorganisms of fresh fruit and vegetables, ready-to-eat vegetables, and fruit juices. 

With regard to the application of plant antimicrobials on fresh fruits and vegetables, pomegranate (*Punica granatum* L.) peel extract (PPE), rich in polyphenols such as punicalagin and ellagic acid, reduced the growth of post-harvest fungi belonging to the genera *Penicillium*, *Botrytis*, *Monilinia*, and *Colletotrichum* on various fruits including lemon, strawberry, grape, apple, grapefruit, orange, and capsicum. In addition, the PPE ethanolic or aqueous extracts can preserve foods by dipping treatments or using edible coatings [117]. In this context, the use of ethanolic pomegranate peel extract (PPE) was found to significantly (*p* ≤ 0.05) reduce the lesion diameter and infection rate in mandarins contaminated with *P. italicum* and *P. digitatum* [118]. In addition, other plant-based extracts were also found to be effective in controlling spoilage microorganisms. A mango kernel extract, rich in mangiferin, chlorogenic acid, and myricetin, inhibited anthracnose development caused by *Colletotrichum brevisporum* on mangoes [119]. A sweet orange (*Citrus sinensis* L.) peel extract, rich in ferulic acid, showed antifungal activity against *M. fructicola* and *Alt. alternata* in a peach-based medium [120].

Other plant antimicrobial extracts with antimicrobial activity against spoilage microorganisms on fresh fruit and vegetables are the essential oils or their main compounds. Mint (*Mentha* × *piperita* L.), basil (*Ocimum basilicum* L.), lavender (*Lavandula angustifolia* Mill.), and thyme EOs in the vapour phase were used for the post-harvest preservation of strawberry, peach, orange, and lemon [121,122,123,124]. In particular, as recently reported by Pinto et al. [123], the in-package application of red thyme oil vapours reduced the percentage of infected wounds, the mycelium development, and the production of spores by *Penicillium* strains on oranges during 12 days of cold storage. Dipping in cinnamon essential oil microemulsion at 0.3% *v*/*v* eradicated *P. fluorescens* from iceberg lettuce during 28 days of cold storage [124]. As regards other plant antimicrobials, methyl, allyl, and ethyl isothiocyanate (8–12 µL L^−1^) completely inhibited citrus sour-rot caused by *Geotrichum citriaurantii* [125], whereas berberine at 3 mg mL^−1^ reduced the development of *P. italicum* and natural decay on citrus fruit [88].

Plant antimicrobials were extensively used to control the spoilage microorganisms on ready-to-eat fruits and vegetables [126]. Dipping of fresh-cut pineapple in *Centella asiatica* extract, rich in quercetin and kaempferol, reduced the *A. niger* load during cold storage [127]. In-package application of trans-anethole in ready-to-eat organic lettuce reduced total coliforms during cold storage [128], whereas the addition of β-caryophyllene-rich pepper EOs in salad dressing decreased *P. fluorescens* development and spoilage activity on fresh-cut lettuce [129]. Pomegranate arils coated with savoury essential oil-loaded chitosan showed a reduction in total mesophilic bacteria and total yeasts and moulds of 1 log CFU g^−1^ after 18 days of storage [130]. Peppermint and tea tree (*Melaleuca alternifolia* Cheel) oils controlled the growth of total aerobic bacteria, yeasts, and moulds on fresh-cut green bean pods stored for 9 days at 5 °C [131]. 

Other applications of plant antimicrobials in plant-based foods concern fruit juices and smoothies [132]. In this context, essential oils and their compounds are the most used antimicrobials. Indeed, *Mentha piperita* L. EO inclusion (7.50 µL mL^−1^) in cashew and guava juice caused >5 log reductions in counts of the spoilage yeast *Pichia anomala* [133]. Thymol in concentrated apple juice showed higher antimicrobial activity than carvacrol and trans-cinnamaldehyde against *Z. rouxii* [134]. Lee et al. [135] found a synergism between oregano and thyme EOs, at 0.156 μL mL^−1^, in inhibiting *Leuconostoc citreum* in tomato juice. As regards the applications of plant antimicrobials in smoothies, the addition of beet (*Beta vulgaris* L.) leaf extract (30% *w*/*v*) in a vegetable smoothie reduced significantly (*p* ≤ 0.05) total mesophilic bacteria, enterobacteria, and total yeasts and moulds throughout 21 days of cold storage [136]. 

The use of plant antimicrobials in plant-based food products, specifically fruit juices and fresh and ready-to-eat vegetables, effectively reduces spoilage and increases the shelf life of these products. Essential oils and their bioactive compounds, such as *Mentha piperita* L. EO, thymol, carvacrol, trans-cinnamaldehyde, oregano and thyme EOs, demonstrated antimicrobial activity against different spoilage microorganisms. These findings suggest that plant antimicrobials have the potential to play a crucial role in preserving the quality of plant-based foods.

#### 3.1.2. Applications in Animal-Based Foods

This section presents some applications of whole plant extracts or their antimicrobial compounds against spoilage microorganisms contaminating animal-based foods (e.g., meat, seafood, and dairy products). In this context, the addition of 200 mg kg^−1^ of tannic acid or catechin in camel meat decreased total mesophilic and psychrophilic bacterial counts by one order of magnitude after 9 days of refrigeration [137], as well as Nowak et al. [138] demonstrated that sour cherry (*Prunus cerasus* Scop.) leaf extract, rich in coumaric acid, and blackcurrant (*Ribes nigrum* L.) leaf extract, rich in gallic acid and quercetin derivatives, delayed the growth of *Pseudomonas* spp. in pork sausages, but not that of *Brochothrix* spp. and *Enterobacteriaceae*. On the contrary, Casaburi et al. [139] reduced the growth of *Brochotrix* spp. and *Enterobacteriaceae*, but not that of *Pseudomonas* spp., of grounded beef meat during cold storage, adding 5% of a freeze-dried myrtle (*Myrtus communis* L.) extract, rich in phenolic compounds. These results highlight that the effectiveness of phenolics can vary depending on the specific bacterial species, the concentration of phenolics, and other factors such as the food matrix, the presence of other preservatives, and the storage conditions.

The addition of the ethanolic extract of cranberry (*Vaccinium oxycoccos* L.) pomace, characterized by great amounts of anthocyanins, chlorogenic acid, and myricetin and quercetin derivatives, inhibited the growth of *Brochothrix thermospacta* and *P. putida* on pork burgers during the first days of cold storage [140]. As regards the application of essential oils or their compounds on meat products, ethanolic extracts of rosemary and clove (1% *v*/*w*) reduced *Pseudomonas* spp. counts in raw chicken meat during cold storage [141]. The use of *Ziziphora clinopodioides* essential oil (0.2% *v*/*w*), rich in carvacrol and thymol, reduced the *Enterobacteriaceae* and psychrotrophic bacteria loads of raw beef patties during cold storage by 2–3 log cfu g^−1^ [142]. Thymol or carvacrol at 0.4% *w*/*w* in marinated beef significantly reduced the mesophilic total viable count, lactic acid bacteria, *Broch. thermosphacta*, *Pseudomonas* spp., and total coliforms, extending the microbiological shelf life by three days [143]. In this context, only some terpene compounds showed a broad spectrum of activity against various bacterial species, making them effective preservatives for meat products, independently of the source of plant origin, and able to extend the shelf life of some meat products.

Likewise, the reduction in fish spoilage bacteria can be achieved using plant antimicrobials, specifically polyphenolic extracts and essential oils [11]. The use of ethanolic Noni (*Morinda citrifolia* L.) leaf extract, rich in rutin and kaempferol derivatives, was shown to extend the shelf life of striped catfish slices and maintain the acceptable levels of total viable bacteria and psychrophilic bacteria during storage, with loads remaining below 6 log cfu g^−1^ [144]. Similarly, the growth of *Pseudomonas* spp. in Pacific white shrimps was delayed by adding ethanolic guava (*Psidium gujava* L.) leaf extracts, rich in phenolic compounds such as piceatannol 4′-galloylglucoside, epicatechin, epigallocatechin, procyanidin B2, ellagic acid, quercetin 3′-o-glucuronide, and quercetin 3-galactoside [145]. Grape seed extract, containing high levels of phenolic acids, catechins, and proanthocyanidins, decreased the presence of *Aeromonas* spp. in snakehead fillets during cold storage. This reduction limited the release of soluble peptides and biogenic amines and increased the shelf life of snakehead fillets by three days [146]. The application of essential oils, such as cinnamon, oregano, and thyme, as marinades was evaluated in salmon and scampi by Van Haute et al. [147]. The immersion of these products in cinnamon essential oil at 1% *w*/*w* inhibited the growth of yeasts and moulds. Similarly, cinnamon essential oil at 0.1% *w*/*v* effectively inhibited *Aeromonas* spp. in vacuum-packed carp and extended its shelf life by two days [148]. However, the direct application of essential oils in fish products can cause bitterness, off-flavours, and yellowing of the tissue [11]. The inclusion of essential oils in active packaging or nano-emulsions is recommended to mitigate these effects.

Building on the findings of previous studies on the application of plant antimicrobials in meat and fish products, the use of plant polyphenols, essential oils, and other plant-based compounds in milk and dairy products to control spoilage microorganisms and extend their shelf life is also of interest. For instance, the addition of olive mill wastewater in the governing liquid of “Fior di Latte” cheese (500 µg mL^−1^ of phenols) resulted in a four-day extension of shelf life due to the increase in the lag phase of *P. fluorescens* and *Enterobacteriaceae* [149]. A recent study by Derbassi et al. [150] evaluated the preservative effect of *Arbutus unedo* L. leaf extracts on the microbiological characteristics of quark cheese during storage. They found that incorporating the dry macerated leaf extract into the cheese resulted in higher efficacy against aerobic mesophiles and yeasts than the use of potassium sorbate after 8 days of storage. Milanović et al. [151] investigated the efficacy of seven essential oils against 74 spoilage yeasts. In a yoghurt model, lemongrass and cinnamon EOs demonstrated the highest antifungal activity in vitro. However, it should be noted that cinnamon EO inhibited lactic acid bacteria, while lemongrass EO displayed species-specific antifungal activity. These findings suggest that further research is needed to fully understand the application of plant antimicrobials in the dairy sector to control spoilage microorganisms.

The direct addition of natural plant antimicrobials in animal-based foods, such as meat, fish, and dairy products, shows the potential to control spoilage microorganisms and extend shelf life. Studies demonstrated the effectiveness of compounds such as phenolic acids, catechins, proanthocyanidins, and EOs in inhibiting the growth of spoilage bacteria. However, more research is necessary to fully understand the mode of action of these natural compounds and optimize their application in animal-based foods. Additionally, it is essential to consider the potential drawbacks, such as the development of off-flavours or bitterness, and address them through alternative delivery methods, such as nano-emulsions or active packaging.

**Table 1:** Applications of plant antimicrobials on plant-based and animal-based foods against spoilage microorganisms.

Food Matrix	Plant Antimicrobial	Concentration/Conditions	Antimicrobial Effect	Data from Ref.*
Mandarins	Pomegranate peel extract	Dipping in 25 g L^−1^ extract for 2 min	Reduction of lesion diameter and infection rate (80–90%) caused by *P. italicum* and *P. digitatum*	[118]
Fresh-cut lettuce	Pepper EO	3–5 µL mL^−1^ addition in salad dressing	Reduction of *P. fluorescens* biomass by 30–40%	[129]
Concentrated apple juice	Thymol, carvacrol	MIC of 0.1–0.16 mM, treatment time 9 days	Reduction of *Z. rouxii* load by 99%	[134]
Pork burgers	Ethanolic extract of cranberry pomace	2% extract-16 days of storage	Bacteriostatic effect on *B. thermospacta* and *P. putida* during cold storage	[140]
Snakehead fillets	Grape seed extract	0.52 mg GAE mL^−1^ for 20 min	Decrease of *Aeromonas* spp. abundance by 37% and reduction of 1 log cfu g^−1^ of total viable counts during cold storage	[146]
Quark cheese	*Arbutus unedo* L. leaf extracts	0.1 g 100 g^−1^ cheese, 8 days of cold storage	Reduction of total aerobic mesophilic bacteria and yeasts by 2–3 log cfu g^−1^	[150]

* as cited in the text.

### 3.2. Use of Plant Antimicrobials for Food Safety

The use of natural compounds derived from plants has numerous benefits, including the potential to provide safer, more sustainable and practical solutions for preserving food safety [152]. These plant-derived compounds showed high antimicrobial activity, making them ideal candidates as natural food preservatives. In particular, the correct use of these natural antimicrobials can fight emerging problems such as the spread of multidrug-resistant pathogens, biofilm-producing strains, and microbial toxins through the food chain.

#### 3.2.1. Effect on Viability of Foodborne Pathogens

Foodborne bacteria are a significant public health concern since they can cause gastrointestinal illness, food poisoning, chronic diseases, economic losses, and the spread of antibiotic-resistant bacteria. Multiple foodborne illnesses were caused by various pathogens such as *Sal. enteritidis*, *L. monocytogenes*, *E. coli* toxigenic strains, *Cam. jejuni*, *Cronobacter sakazakii*, and *S. aureus.* Foodborne outbreaks underline the need for more efficient methods to control foodborne pathogens. Symptoms of foodborne illness can range from mild to severe, including nausea, vomiting, diarrhoea, abdominal cramps, and fever [153]. The outbreak of foodborne illnesses can have significant economic consequences, including loss of income for food producers, increased healthcare costs, and decreased consumer confidence in the food industry. Addressing the issue of foodborne bacteria is crucial to ensure the safety and quality of the food supply, protect public health, and minimize the economic impact of foodborne illnesses.

Several phytochemicals showed antibacterial activity against various foodborne pathogens. For example, studies demonstrated that plant compounds such as carvacrol and thymol, found in essential oils extracted from herbs and spices, have high antibacterial activity against *Sal. enteritidis*, *E. coli*, and *L. monocytogenes* [154]. Similarly, compounds such as cinnamaldehyde and eugenol, present in cinnamon EO and clove EO, respectively, inhibited the growth of foodborne pathogens such as *L. monocytogenes* and *S. aureus* [155,156]. These findings provide evidence of the potential of plant-based antimicrobials in controlling foodborne pathogens and improving food safety.

Specific applications of EOs or their compounds were described in plant and animal-based foods to ensure food safety. As regards animal-based foods, cinnamaldehyde inactivated *L. monocytogenes* at 4 °C in ground pork, reducing its viability by 4 log cfu g^−1^ in 5 days [155]. Similarly, thymol reduced, by 3 log cfu g^−1^, the load of *S. aureus*, *E. coli*, and *C. perfringens* on a sausage product during 4 weeks of storage [157]. In dairy products, myrtle EO (31.25 μL mL^−1^) reduced, by 1–2 log cfu g^−1^, the load of *L. monocytogenes* ATCC 679 on sheep cheese during ripening [158], whereas ginger (*Zingiber officinale* R.) and thyme EOs totally inactivated *S. aureus* (6 log cfu g^−1^) on a fresh soft cheese after two weeks of storage [159]. In plant foods, EOs or their compounds were proposed as sanitizers of fresh-cut vegetables and natural preservatives of fruit juices. Rossi et al. [160] treated fresh-cut lettuce contaminated with a cocktail of *Salmonella* spp. strains, with 5 µL mL^−1^ of cinnamon EO, reducing the attached cells by 0.6–0.8 log cfu cm^−2^. Cinnamon EO was also successfully used to control *Sal. typhimurium* and *L. monocytogenes* on celery, with a reduction of 2–4 orders of magnitude after 7 days at 4 °C depending on the initial contamination level [161]. As regards the application of EOs in fruit juices, *Litsea cubeba* Pers. EO reduced 3–4 log cfu mL^−1^ of the load of *E. coli* O157:H7 in four vegetable juices after 4 days of storage, and inhibited the respiratory metabolism, the topoisomerase activity, the transcription of virulence genes, and the nucleic acid replication [162]. In watermelon juice, *Melissa officinalis* L. EO reduced the viability of *L. monocytogenes* from 2 to 7 days of storage [163]. In some cases, plant antimicrobials can induce tolerance to environmental stresses in bacteria, and cross-resistance to common antibiotics. The use of *Melissa officinalis* L. EO at subinhibitory levels (0.125 μL mL^−1^) did not induce high tolerance to stresses (such as high temperature, low pH, osmotic stress, and desiccation) or cross-resistance with antibiotics in *L. monocytogenes* [163]. 

Plant phenolic compounds are naturally occurring compounds found in plants used as food preservatives due to their high antimicrobial activity against foodborne pathogenic bacteria [92]. Some of the most commonly used plant phenolic compounds in food include quercetin, and derivatives of cinnamic acid and gallic acid. Grape skin pomace extracts from different cultivars, rich in phenolic acids and flavonoids, showed higher antibacterial activity against Gram-positive strains than Gram-negative ones [164]. The addition of cranberry pomace extracts, rich in quinic and chlorogenic acids, procyanidin B3, myricetin and quercetin derivatives, delayed the growth of *L. monocytogenes* in cooked ham during cold storage [144]. *Yersinia enterocolitica* load was reduced by two logarithmic cycles in pork meat containing 5 mg g^−1^ of gallic acid [165]. Phuong et al. [166] evaluated the antibacterial activity of rambutan (*Nephelium lappaceum* L.) peel extracts, rich in geraniin, ellagic acid, rutin, quercetin, and corilagin as main phenolic compounds. The phenolic extract inhibited the growth of *Sal. Enteritidis* in raw chicken and that of *Vibrio parahaemolyticus* in fish during cold storage. The application of polyphenolic extracts or single polyphenols reduced the growth of foodborne pathogens in fresh-cut fruits, as demonstrated by using pomegranate peel extract or ferulic acid against *L. monocytogenes* on fresh-cut pear, apple, and melon [167,168]. The dipping of fresh-cut potatoes and fresh-cut lettuce in *Centella asiatica* L. extract significantly reduced the load of *B. cereus* and *E. coli* O157:H7 [131]. The glabridin, a prenylated isoflavonoid, reduced, by at least 1 log cfu g^−1^, the load of *L. monocytogenes* on fresh-cut cantaloupe during 4 days of cold storage [169]. 

As regards the application of the glucosinolate derivatives against food pathogens, the (4-[(4′-O-acetyl-α-l-rhamnosyloxy)benzyl] isothiocyanate) from *Moringa oleifera* seeds reduced the viable load of *Cro. sakazakii* and *B. cereus* in goat milk by three orders of magnitude [170]. 

In addition to the effect on cell viability, plant antimicrobials improved the thermal sensitivity of foodborne pathogens in the food matrix. In particular, the use of oregano EO in combination with citric acid enhanced the thermal inactivation of *L. monocytogenes* in sous-vide salmon cooked at 60 °C [171], whereas vanillin and emulsified citral improved the heat-sensitization of *E. coli* at 58 °C in a blended carrot-orange juice [172]. However, in certain conditions, plant antimicrobials can induce a viable but not culturable (VBNC) state in foodborne pathogens, as demonstrated for the application of citral and *trans*-cinnamaldehyde in a meat-based broth against *S. aureus* [173]. 

In contrast with many of the above-reported papers, the methanolic extract of spices mixtures employed to confer typical pungency and a hot taste to ‘Nduja, a traditional Calabrian sausage produced with about 20% of different spices, showed a limited inhibitory spectrum against ten common foodborne bacteria. Authors concluded that these spice mixtures, rich in hundreds of potentially antimicrobial compounds, can not exert an antimicrobial effect under normal processing conditions, due to the limited release of the bioactive compounds from the plant tissue [174]. In conclusion, the inclusion of plant antimicrobials in real food model systems can control the growth of foodborne pathogens, representing a valuable option to replace synthetic preservatives, even though their efficacy needs to be carefully evaluated under real production conditions. 

#### 3.2.2. Effect on Biofilm-Producing Strains

Bacterial biofilms are communities of microorganisms encased in a self-produced extracellular matrix and attached to a surface [22]. Biofilms are prevalent in many natural and artificial environments, including food processing facilities and equipment. Bacterial biofilms can cause serious problems in the food industry by contaminating food products, leading to foodborne illness and decreasing the product’s quality [115]. Biofilms can harbour pathogenic bacteria and provide a protective environment for these microorganisms, making them resistant to cleaning and disinfection procedures. This can result in a persistent contamination and the spread of foodborne illnesses. In addition, biofilm growing on the equipment surfaces can cause clogging, formation of corrosion, and degradation of the equipment surfaces, leading to increased maintenance costs and decreased productivity [175]. Bacterial biofilms are a significant concern in the food industry due to their impact on food safety and quality and the performance and efficiency of food processing equipment. The food industry needs to implement effective strategies to prevent and control the formation of bacterial biofilms to maintain a safe and efficient food processing environment.

The use of plant-derived antimicrobial compounds in food preservation gained attention due to their efficacy against foodborne biofilm-producing strains of bacteria [176]. These compounds act through various mechanisms, such as interference with metabolic processes, oxidative stress, and membrane disruption, and can also exert positive effects in inhibiting the growth and replication of biofilm-producing bacteria. The first anti-biofilm mechanism of action is the inhibition of the bacteria’s attachment to the surfaces. Phenolic compounds, such as phenolic acids, catechins, and quercetin, were found to reduce the adhesion of bacteria affecting flagellum, fimbria, and adhesins, delaying the formation of biofilms [12]. Red Globe and Carignan grape stem extracts, rich in caffeic, ferulic and gallic acids, catechin and rutin, inhibited the adhesion of *L. monocytogenes* to stainless steel and polypropylene surfaces by inhibiting motility and reducing the adhesion potential [177], as well as quercetin inhibited the early attachment of *L. monocytogenes* on stainless steel surface by increasing the cell permeability and reducing the superficial cell charge [178,179]. Quercetin also reduced the swimming and swarming motility of *Sal. enterica* at sub-MIC levels [180].

EOs or their compounds inhibit biofilm formation by different mechanisms. Cinnamon EO inhibited the adhesion of *L. monocytogenes* on polystyrene, but its efficacy was low on pre-formed biofilm [22]. Additionally, some terpenes, such as eugenol, carvacrol, and thymol, were demonstrated to suppress the production of exopolysaccharides in *Salmonella* spp., which are key components of bacterial biofilms [175], whereas citral and geraniol decreased the glucan production in *E. coli* O157:H7 [181]. Eugenol showed similar effectiveness against sessile and planktonic cells of *S. aureus*, showing a lower resistance coefficient, the ratio of concentrations required to achieve the same log reductions in both populations (C_biofilm_/C_planktonic_), as compared to conventional disinfectants [182]. Carvacrol and oregano EO effectively inhibited biofilm formation by *S. aureus* on stainless steel surfaces, but the long-term exposure to a sub-MIC concentration of the oregano EO showed an inductive biofilm formation effect [183]. Another mode of action of plant antimicrobials against foodborne biofilm-producing bacteria is destabilizing the biofilm matrix. Compounds such as sulphides, including allicin and diallyl sulfide, and sulfites were shown to penetrate the biofilm and disrupt its stability, causing the release of bacteria from the biofilm [184], as demonstrated in uropathogenic *E. coli* [185].

On the other hand, plant-derived antimicrobial agents were shown to possess anti-biofilm activity by disrupting the quorum sensing process [186]. Quorum sensing is a communication mechanism that bacteria utilize to coordinate the expression of certain genes, including those involved in biofilm formation. Phytochemicals such as flavonoids (quercetin and kaempferol) and terpenoids (carvacrol and thymol) were demonstrated to interfere with the quorum-sensing by inhibiting the production and the activity of autoinducers (e.g., acyl-homoserine lactone), which play a key role in the quorum-sensing process [187,188]. *Lippia origanoides* K. EO (thymol-carvacrol chemotype) inhibited the expression of the *sdiA*, *luxS*, and *luxR* genes, which were implicated in the quorum-sensing of *Sal. enteritidis*. This effect could be related to the inhibition of the biosynthesis of autoinducers or the interference with the reception of acyl-homoserine lactone [189]. Aqueous pomegranate extract showed anti-quorum sensing activity, reducing the violacein production, the quorum-sensing system’s product, in *Chromobacterium violaceum* [186]. *Curcuma longa* L. extract, with curcumin and curcumin derivatives as main compounds, showed anti-quorum sensing activity inhibiting the violacein production in *C. violaceum*, probably disrupting the signal reception or the absorption of the acyl-homoserine lactone. However, this extract showed lower anti-biofilm activity against food pathogens than *Camellia sinensis* L. extract, rich in epigallocatechin and epicatechin [190].

**Figure 2:**
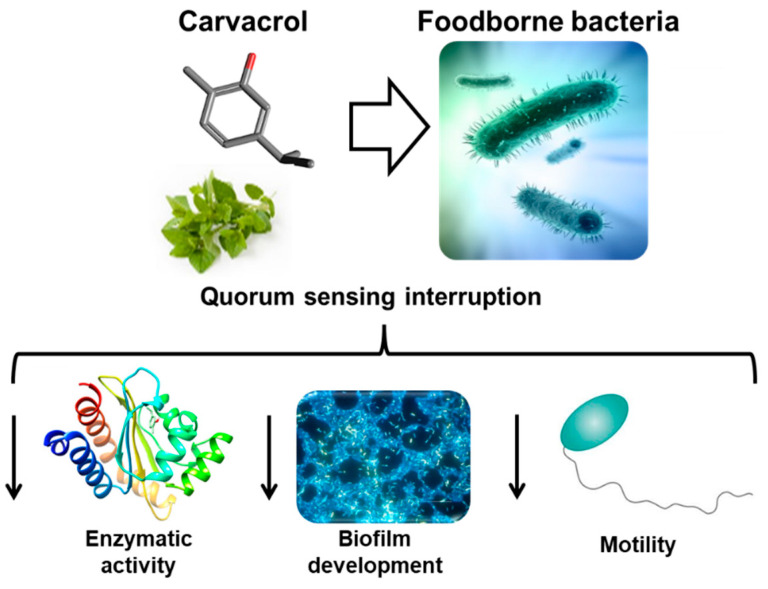
Anti-quorum sensing potential of carvacrol in foodborne bacteria.

The use of plant antimicrobials as a strategy to control the biofilm formation in foodborne pathogens gained increasing attention in recent years. Phytochemicals such as phenolic acids, tannins, sulphur compounds, and terpenoids (Figure 2) showed anti-biofilm activity by interfering with the quorum-sensing process of bacteria [191]. Despite these promising results, further research is needed to fully understand how these compounds exhibit antibacterial and anti-biofilm activity and to develop effective strategies for controlling biofilm formation during food processing. 

#### 3.2.3. Effect on Microbial Toxins 

Microbial toxins (e.g., bacterial toxins and mycotoxins) harm human health. Plant antimicrobials were evaluated to reduce toxin production by foodborne bacteria and mycotoxins by filamentous fungi. 

Bacterial exotoxins are proteins that damage host cells and are important for the pathogenesis of many bacterial pathogens, such as *Clostridium* spp., *E. coli*, *L. monocytogenes*, and *S. aureus* [192]. The use of plant antimicrobials can attenuate the virulence of these foodborne pathogens. In particular, different flavonoids suppressed the toxin production in different foodborne pathogens. Genistein inhibited the exotoxin produced by *S. aureus*, kaempferol, kaempferol-3-O-rutinoside, quercetin glycoside inhibited the neurotoxin production from *Cl. botulinum*, and green tea catechins inhibited the release of verotoxin from enterohemorrhagic *E. coli* [16]. Recent findings showed that the water-soluble fraction of the *Eucalyptus camaldulensis* Dehnh. leaf extract significantly reduced the listeriolysin O-induced haemolysis in *L. monocytogenes* at sub-inhibitory concentrations [193]. A witch-hazel extract, with hamamelitannin as the main phenolic compound, inhibited the production of the staphylococcal enterotoxin A in *S. aureus* at non-inhibitory concentrations for microbial cells [194]. As regards the EO and their compounds, sub-inhibitory concentrations of tea tree EO downregulated the transcription of genes encoding α-hemolysin, staphylococcal enterotoxin A, and staphylococcal enterotoxin B in *S. aureus*, inhibited their production, and the hemolytic activity [195]. Zhang et al. demonstrated that citronellal significantly reduced the production of enterotoxins in *S. aureus*-contaminated pork meat without reducing the viable cell load [196]. Other EO compounds, such as carvacrol and trans-cinnamaldehyde, reduced the production of TcdA and TcdB toxins produced by *Cl. difficile* in in vitro conditions [197]. Organic sulphur compounds such as the diallyl disulphide, at sub-inhibitory concentrations, reduced the production of the *B. cereus* enterotoxins Nhe and Hbl [91]. 

Plant antimicrobials also showed the ability to control the mycotoxin production by filamentous fungi. The mechanisms of action are the inhibition of the fungal growth and the induction of xenobiotic detoxification and/or the activation of biotransformation pathways [19]. In the first case, turmeric, rosemary and clove EOs demonstrated great efficacy in controlling the growth of mycotoxigenic *A. flavus* through the inhibition of ergosterol biosynthesis, the disruption of the fungal cell membrane, and the production of reactive oxygen species (ROS). In some cases, essential oils showed anti-aflatoxigenic activity at concentrations inhibiting or completely suppressing fungal growth. In contrast, in other cases, the anti-aflatoxigenic activity was detected at non-inhibiting concentrations. However, in a few cases, plant antimicrobials stimulated the production of secondary metabolites, including mycotoxins, in *Aspergillus* species [19]. Natural flavonoids such as baicalein, flavone, hispidulin, kaempferol, and liquiritigenin reduced the aflatoxin production in maize kernels contaminated with *A. flavus* by 50–67% [198], whereas a ternary mixture of naringin, neohesperidin, and quercetin reduced the aflatoxin accumulation in maize contaminated with *A. parasiticus* by more than 85% [199]. In sausages, the combined application of *Salvia farinacea* Benth. and *Azadirachta indica* A.Juss. extract at 2 mg mL^−1^ suppressed the production of ochratoxin A and aflatoxin B1 produced by *A. ochraceous* and *A. parasiticus*, respectively [200]. The degradation of aflatoxin B1 treated with the leaf extract from rosemary reached 60% after 48 h of incubation. Araçá (*Psidium cattleianum* S.) and oregano extracts produce less degradation than rosemary extract. Substances such as alkaloids and enzymes occurring in the plant extract might be involved in the structural modification of aflatoxin B1 [201]. Although the effect of plant antimicrobials on mycotoxin accumulation in food products is promising, more in-depth information regarding the toxicity of the resulting compounds from the degradation activity is required. 

In conclusion, plant antimicrobials can reduce or suppress the production of bacterial and fungal toxins by reducing microbial growth or downregulating toxin gene expression. Further research is necessary to understand the modes of action of different plant extracts and their bioactive compounds on toxin production to exploit their potential to improve food safety under real contamination conditions.

## 4. Stabilization Techniques

Plant antimicrobials can have limited stability under processing or storage conditions of foods. The efficacy of plant antimicrobials is affected by several factors such as pH, the temperature, and the concentration. Caffeic, chlorogenic, and gallic acids are not stable at high pH values, whereas chlorogenic acid is stable at low pH values and heat [202]. Some phenolic compounds and EOs, and their compounds, are thermolabile. *Achillea* sp., rosemary, sage (*Salvia officinalis* L.), and thyme EOs were more effective at low pH and low temperature against pathogenic bacteria [203], whereas carvacrol and cymene showed higher antibacterial activity in carrot juice at 25 °C than at 4 °C and 15 °C [204]. Several plant antimicrobials show a dose-dependent effect against spoilage and pathogenic microorganisms. The stabilization techniques described in this section can help to protect plant antimicrobials and, in some cases, reduce the concentration necessary to exert their antimicrobial activity. The direct addition of plant extracts or their bioactive compounds in foods is the most common method of food preservation. However, the direct addition of plant extracts is often responsible for changes in sensory properties such as flavour and texture. In addition, the bioavailability of these compounds and their effectiveness in improving food safety can be affected by the interaction with the macronutrients and ingredients. For these reasons, several stabilization techniques were proposed to enhance stability, drive the release of bioactive compounds during storage, and reduce the negative effects of plant extracts on the sensory characteristics of foods.

### 4.1. Nano-Emulsions

The encapsulation of plant antimicrobials into edible colloidal delivery systems is a promising method to enhance the efficacy of these substances and reduce the negative effects due to the interaction with food ingredients. In particular, encapsulation in small particles increases water dispersibility and resistance to environmental conditions enhancing plant antimicrobials’ efficacy [6]. Oil-in-water nano-emulsions containing lipid nanoparticles dispersed in water are currently the most common delivery system for plant antimicrobials. These nano-emulsions can be manufactured from food-grade ingredients, such as plant-based emulsifiers and different stabilizers, using common processing methods, such as mixing (low-energy emulsification), sonication, and homogenization (high-energy emulsification) [6]. 

Different studies investigated the efficacy of nano-emulsions against foodborne pathogens. The plant antimicrobials most used to prepare nano-emulsions are the EOs and their compounds. Lemongrass, clove, thyme, or palmarosa (*Cymbopogon martini* Will. Watson)-loaded EOs nano-emulsions, prepared after micro fluidization of the primary emulsion, inactivated *E. coli* by 3–4 log cfu mL^−1^. The use of alginate in the aqueous phase is useful for applying these nano-emulsions in the coating material of fruits and vegetables [205]. Anise (*Pimpinella anisum* L.) oil nano-emulsions showed the same MIC (1% *v*/*v*) of the bulk EO and coarse emulsion against *L. monocytogenes* and *E. coli* O157:H7. However, the anise oil nano-emulsion displayed the highest physical stability and antibacterial efficacy [206]. More recently, other plant antimicrobials were used to prepare nano-emulsions to control the growth of bacterial pathogens. Anise seed extract, with anethole, naringenin, and taxifolin as main compounds, was used to develop an antibacterial nano-emulsion using the ultrasound emulsification method. The nano-emulsion was active against *E. coli* and *Sal. thyphimurium*, whose growth was not affected by the bulk extract [207]. Ghazy et al. [208] evaluated the antimicrobial action of henna (*Lawsonia inermis* L.) extract as a nano-emulsion against seven pathogenic bacteria. The nano-emulsion, rich in catechin, methyl gallate, ellagic acid, and coumaric acid, displayed higher antimicrobial activity against *E. coli*, and *B. cereus*, than the course emulsion. Regarding the application of nano-emulsions, including plant essential oils, to control pathogens in plant foods, oregano oil nano-emulsion at 0.1% reduced the load of *L. monocytogenes*, *Sal. typhimurium*, and *E. coli* O157:H7 on lettuce by 3 log cfu g^−1^ [209]. Cinnamon oil nano-emulsion at 0.5% determined more than five log reductions in *L. monocytogenes* and *Salmonella* spp. on melon [210]. Lemongrass and mandarin (*Citrus reticulata* Blanco) EO nano-emulsions inactivated *E. coli* in apple juice, but when the nano-emulsions were prepared directly in the apple medium as a continuous phase, the antibacterial efficacy was reduced in comparison to the use of water [211]. Citral nano-emulsions at 0.15 μL mL^−1^ inactivated *L. monocytogenes* (5 log cfu g^−1^ reduction) on fresh-cut melon and papaya during cold storage [212].

Regarding the efficacy of nano-emulsions including plant antimicrobials against spoilage microorganisms, thyme EO nano-emulsion showed lower efficacy than bulk EO against fish spoilage bacteria, except for *Serratia liquefascens* [213]. For this spoilage bacteria, laurel (*Laurus nobilis* L.) and grapefruit (*Citrus paradisi* Macfad.) EO nano-emulsions showed lower MIC values than the corresponding EOs [214,215]. Ginger EO nano-emulsion, prepared with zein and sodium caseinate as co-emulsifiers, showed higher bactericidal activity against total viable counts of chicken breasts than the bulk EO, extending the shelf life of the product by 6 days [216]. As regards the antifungal activity of plant antimicrobial nano-emulsions, cinnamaldehyde, eugenol, and carvacrol nano-emulsion showed a dose-dependent effect against the spore germination and mycelial growth of *P. digitatum*, with a MIC value of 0.125 mg mL^−1^ [217]. Gundewadi et al. [218] found that basil EO nano-emulsion displayed lower lethal concentration values (LC_50_) than course emulsion against *P. chrysogenum* and *A. flavus* during 8 days of incubation. Oregano and clove EOs nano-emulsions, at 1.95 mg g^−1^, showed fungicidal activity against *Z. bailii* in a salad dressing after 4 days of storage [219]. EOs nano-emulsions also showed anti-mycotoxigenic activity. Indeed, lemongrass EO nano-emulsion reduced by 99.5% the deoxynivalenol content in rice contaminated with *F. graminearum*. The lemongrass EO nano-emulsion showed better anti-mycotoxigenic activity than the bulk EO, but the efficacy was strain-specific [220]. Oregano EO encapsulated into chitosan nano-emulsion suppressed the production of aflatoxin B1 by *A. flavus* in maize [221].

Given these results, the antimicrobial action of plant antimicrobial nano-emulsions depends on the chemical composition of the plant extract, the emulsion droplet size, and the target microbial species. In addition, many studies demonstrated higher efficacy of nano-emulsion than course emulsion and bulk plant extract.

### 4.2. Spray-Drying and Encapsulation

Spray-drying and encapsulation are techniques commonly used to improve the stability and functionality of plant antimicrobials in food products. Spray-drying is a process in which a liquid solution or suspension is atomized into a hot air stream, causing the rapid evaporation of droplets, resulting in a dry powder. This process can produce dry powders of plant antimicrobials that are more stable and easier to handle than the liquid form. Spray-drying can also encapsulate the plant antimicrobials in a protective matrix, improving their stability and functionality. Encapsulation is a process in which a natural antimicrobial is surrounded by a protective matrix, such as a polymer or lipid, to improve its stability and functionality. Encapsulation can enhance the natural antimicrobials’ shelf life and protect them from degradation induced by light, heat, or moisture. Additionally, encapsulation can improve the solubility and dispersibility of natural antimicrobials, making them easier to incorporate into foods. These techniques can help to preserve the antimicrobial activity of the natural antimicrobials and improve their effectiveness in controlling the growth of spoilage and pathogenic microorganisms.

#### 4.2.1. Spray-Drying Process 

Spray-drying and encapsulation techniques provides numerous benefits in handling, storage, and transportation of plant antimicrobials. Powdered antimicrobials are more suitable for various applications within the food industry [222]. Powdered antimicrobials minimize the risk of spillage and waste during handling and processing, as they can be easily measured and transferred without causing mess or loss of material. This ensures a more efficient use of resources and reduced operational costs. In some cases, the spray-drying process conditions can have pros and cons related to the stability and functionality of plant extracts, as briefly pointed out in Table 2.

In the study of Chen et al. [223], eugenol and thymol were co-encapsulated into zein-casein nano-capsules through spray-drying. The resulting powders showed good water hydration, stability during storage, controlled release during 24 h, and bactericidal and bacteriostatic effects against *E. coli* O157:H7 and *L. monocytogenes* in milk whey, respectively. Thyme EO encapsulated by spray-drying, with casein and maltodextrin as wall materials, showed antibacterial action against thermotolerant coliforms and *E. coli* in meat burgers [228]. The wall material employed to protect plant antimicrobials can affect their antibacterial action. Indeed, the inclusion of chitosan in a whey protein/maltodextrin blend reduced the antibacterial action of eugenol against *E. coli* and *L. innocua*. A low inlet temperature used in the spray-drying of pectin/sodium alginate capsules including carvacrol, increased the antibacterial activity against *E. coli* K12 (Table 2). The use of nano spray-drying, a novel process to produce plant antimicrobial powders, was evaluated to obtain whey protein/maltodextrin capsules, including oregano EO. The capsules showed enhanced antibacterial action against *E. coli* and *S. aureus* compared to pure EO. However, this process has drawbacks such as high production costs, high processing time, and reduced spraying effectiveness of viscous solutions [226]. These examples illustrate the potential benefits and drawbacks of the spray-drying technique to produce plant antimicrobial powders. The specific advantages and disadvantages observed depend on the antimicrobial compound, the spray-drying conditions, and the choice of the protective matrix.

The increased stability of powdered antimicrobials extends their shelf life, as demonstrated for the encapsulated peanut (*Arachis hypogaea* L.) skin extracts [229]. It maintains the efficacy of plant antimicrobials throughout storage, reducing the need for frequent replacements and ensuring consistent antimicrobial action. 

Powdered antimicrobials generally have lower storage requirements than their liquid counterparts, as they do not require refrigeration or specific storage conditions to maintain their stability. This reduces energy consumption and storage costs for food manufacturers. Additionally, the nature, weight, and form of powdered antimicrobials facilitates more efficient transportation and shipping, as they occupy less space and require less protective packaging than liquid antimicrobials [222]. Powdered antimicrobials can be more easily integrated into various food matrices, as their fine and uniform particles allow a more homogeneous distribution throughout the product. This ensures consistent antimicrobial protection across the whole food matrix, enhancing food safety and quality. 

#### 4.2.2. Other Encapsulation Techniques of Plant Antimicrobials

Encapsulation of plant antimicrobials, a method of entrapment of a core material within another solid or liquid immiscible substance, allows the production of capsules or spheres in micrometre to millimetre in size [230]. Encapsulation can involve various types of protective matrices that impact the stability and functionality of the antimicrobial agents. Some common matrices for encapsulation include polysaccharides, lipids, and proteins. Polysaccharides such as alginate, chitosan, and maltodextrin are widely used as encapsulating agents due to their biocompatibility, non-toxicity, and excellent film-forming properties. A study by de Araújo et al. [231] demonstrated that using maltodextrin/gelatine mixtures as a protective matrix for encapsulating the sweet orange EO positively affected the thermo-oxidative stability of bioactive compounds and maintained its antibacterial properties. The encapsulation of plant antimicrobials can be obtained through the formation of inclusion complexes using the β-cyclodextrins, cyclic oligosaccharides with amphipathic properties. These complexes can stabilize the guest molecule against the degradation, mask off-flavours, and control the release of the encapsulated compounds [232]. Thyme EO microcapsules exerted a bacteriostatic effect over *Enterobacteriaceae*, mesophilic bacteria, and psychrotrophic bacteria on lettuce [232], whereas inclusion complexes with rosemary EO showed better antimicrobial activity against *Saccharomyces pastorianus* than free EO in pasteurised tomato juice [233]. Coriander (*Coriandrum sativum* L.) EO encapsulated in β-cyclodextrin nano-sponge showed bactericidal activity against *L. monocytogenes*, *Y. enterocolitica*, and *Cam. jejuni* in aqueous media [234]. Black pepper (*Piper nigrum* L.) oleoresin was stabilized in β-cyclodextrins using the kneading method, a method in which the β-cyclodextrins and the guest compound are mixed with small amounts of ethanol or water using a kneader for a specific time, showing antimicrobial activity against *L. monocytogenes* and improved thermal stability [235].

Lipid-based encapsulation systems, such as solid lipid nanoparticles, the use of nanostructured lipid carriers, and liposomes, are also employed for encapsulating plant-derived antimicrobials. These systems can improve the stability of the encapsulated compounds, their bioavailability, and ensure a controlled release. The study by Lin et al. [236] reported that encapsulating chrysanthemum (*Chrysanthemum flosculosum* L.) EO in triple-layer liposomes led to long-term antimicrobial activity against *Cam. jejuni* in chicken.

Protein-based matrices, such as gelatine, soy protein, and whey protein, can also encapsulate plant-derived antimicrobials. These matrices offer advantages in biodegradability, biocompatibility, and the ability to form stable complexes with antimicrobial agents. Recently, the microencapsulation of cinnamon EO using chitosan and whey protein isolate showed enhanced thermal stability and long-term antimicrobial effect against *S. aureus*, *E. coli*, *P. fragi*, and *Shewanella putrefaciens* [237]. The selection of the most suitable matrix depends on the specific antimicrobial compound, the target application, and the desired release characteristics.

#### 4.2.3. Challenges Associated with Spray-Drying and Encapsulation Techniques 

Although spray-drying and encapsulation techniques offer various advantages for the stabilization and incorporation of plant-derived antimicrobials into food systems, there are challenges associated with these processes that require further research. One of the issues associated with spray-drying is the potential degradation or loss of activity of heat-sensitive plant compounds during the drying process, as high temperatures are often involved [222]. This can lead to reduced antimicrobial efficacy or the modification of sensory properties. Additional and specific research studies need to be carried out to explore alternative drying techniques, such as freeze-drying or nano spray-drying, that might better preserve heat-sensitive compounds. Another challenge is the selection of the most appropriate encapsulation matrix to ensure optimal protection, release, and stability of the encapsulated antimicrobial compound. The choice of the encapsulation material can greatly affect the effectiveness and shelf life of the antimicrobial agent for food applications [238]. Further research is needed to understand the interactions between various wall materials and plant-derived antimicrobials, and to optimize the encapsulation processes for specific food systems. Additionally, scaling up from lab-scale to industrial-scale production of encapsulated plant-derived antimicrobials poses challenges related to the encapsulation efficiency, the product stability, and the process economics [239]. More research is also required to develop cost-effective and efficient methods for large-scale production, maintaining the quality and functionality of the encapsulated antimicrobials. 

### 4.3. Active Packaging

Active packaging involves the deliberate inclusion of subsidiary constituents in or on either the packaging material or the package headspace to enhance the performance of the package system. Active packaging can preserve the food quality and can extend the product’s shelf life through the direct interaction between the food and bioactive substances intentionally incorporated into the package [240]. Antimicrobial packaging is one type of active packaging, in which the antimicrobial activity strongly depends on the migration rate of the biologically active molecule incorporated into the polymer matrix [240]. Recently, the market was oriented to replace packaging produced with fossil fuels with more sustainable materials such as biopolymers. In this context, the use of biopolymers, including plant antimicrobials, is very attractive to develop active films or coatings, to overcome the thermal/oxidative instability of these compounds during the manufacturing of the polymers or storage of the final product, and to mask undesirable sensorial aspects of some plant extracts. In addition, biopolymers have a lower environmental impact compared to standard food packaging polymers such as polyethylene or polypropylene. Plant antimicrobials can be added directly into the biopolymer or loaded into clays or nanocarriers, as demonstrated for EO compounds [241,242] and polyphenols [243,244]. This strategy ensures, in most cases, their controlled release and, in some cases, an improvement in the mechanical and physical properties of the film.

The most used biopolymers for including plant antimicrobials are chitosan, starch, carrageenan, cellulose, and alginate. However, other polymers used for this purpose are polyvinyl alcohol (PVA), poly lactic acid (PLA), poly butylene-succinate-co-adipate (PBSA), poly butylene-adipate-co-terephthalate (PBAT), poly(hydroxybutyrate)s (PHBs), and poly (ε-caprolactone) (PCL).

Regarding active chitosan films, the inclusion of apple peel polyphenols (1%) into chitosan film enhanced the antibacterial activity against *B. cereus*, *E. coli*, *Sal. typhimurium*, and *S. aureus* [245]. A composite film based on grapefruit seed extract-loaded poly(ε-caprolactone)/chitosan reduced the *E. coli* population on salmon by more than 2 log cfu g^−1^ after 6 days at 4 °C compared to the packaging into polyethylene or poly(ε-caprolactone)/chitosan films, and suppressed mould development on bread stored for 7 days at 24 °C [246]. Surendhiran et al. [247] developed active nanofibers based on chitosan/Poly (ethylene oxide) loaded with pomegranate peel extract. The nanofibers reduced by 3 log cfu g^−1^ the *E. coli* O157:H7 population in raw beef stored at 4 °C for 10 days. The coating of fresh cucumber with chitosan loaded with oregano EO reduced the viability of total mesophilic bacteria and total yeasts and moulds during storage at 10 °C for 15 days [248]. Starch films were also enriched with plant antimicrobials as demonstrated by Saberi et al. [249] that developed pea starch-guar gum films including epigallocatechin-3-gallate and blueberry ash fruit (*Elaeocarpus reticulatus* Sm.) and macadamia (*Macadamia tetraphylla*) skin extracts. Active films showed antimicrobial activity against spoilage bacteria and fungi, and pathogenic bacteria, with a reduction in microbial load in the range of 40–80% for the films loaded with epigallocatechin-3-gallate and blueberry ash fruit skin extracts at the MIC level (ranging from 93 to 1500 μg mL^−1^). A bio-composite film made with cassava starch and whey protein loaded with rambutan peel extract and clove oil slightly inhibited *B. cereus*, *E. coli*, and *S. aureus* in in vitro conditions, and reduced total viable count of salami stored for 10 days [250]. A sweet potato starch-based film activated with montmorillonite nano-clay and thyme EO reduced the load of *E. coli* and *S. thypimurium* on fresh spinach leaves during 8 days of cold storage [251]. Other biopolymers used to manufacture active films/coatings with plant antimicrobials are alginate and carrageenan. A sodium alginate film loaded with the gallnut extract (*Quercus infectoria* Oliv.), rich in gallotannins, ellagic acid, and gallic acid, showed antibacterial activity against *S. aureus* and *E. coli* [252], whereas the coating of apples and pears with alginate loaded with cinnamon EO at 0.9% *v*/*v* inhibited the *A. carbonarius* growth and the OTA production [253]. Compared with the control film, a carrageenan film containing 3% rosemary extract displayed >99% inhibition against *B. cereus*, *E. coli*, *P. aeruginosa*, and *S. aureus*, reducing by 2–4 orders of magnitude the microbial load [254]. He and Wang [255] recently demonstrated that a K-carrageenan coating enriched with cinnamon EO delayed the growth of total viable count, lactic acid bacteria, and H_2_S-producing bacteria in pork meat. Active films incorporating plant antimicrobials can be also produced using proteins. Indeed, zein nanofibers loaded with 1,8-cineol rich extracts reduced the load of *L. monocytogenes* and *S. aureus* on cheese slices by two orders of magnitude during 28 days of cold storage [256]. 

Other active films, including plant antimicrobials, can be manufactured using PLA and PHBs. A PLA/PBAT composite film including 7% *w*/*w* of grapefruit seed extract showed bactericidal activity against *L. monocytogenes* and a bacteriostatic effect against *E. coli* [257]. Additionally, a PLA/PBSA blend including 6% *w*/*w* thymol delayed the mould development on bread compared to polypropylene or neat PLA [258]. PHBV films loaded with eugenol and carvacrol showed antibacterial action against *E. coli* in cheese and pumpkin but not in melon, where the highest release of the active compounds from the films was observed [259]. This result highlights that the interaction of antimicrobial compounds with the food components and their diffusion within the food matrix play an important role in the antimicrobial activity of active films including plant antimicrobials. 

Plant antimicrobials can be incorporated into active packaging using encapsulated extracts or nano-emulsions. Pabast et al. [260] developed a chitosan film in which the EO extracted from *Satureja khuzestanica* J. was encapsulated into nanoliposomes. The film delayed the growth of total mesophilic bacteria, *Pseudomonas* spp., and lactic acid bacteria of lamb meat stored for 20 days at 4 °C. Interestingly, the antimicrobial effect was higher than that displayed by the film including free *Satureja khuzestanica* J. EO [260]. Chitosan film loaded with microcapsules, including basil EO, slightly reduces total mesophilic bacteria, enterobacteria, and lactic acid bacteria on cooked ham during storage [261]. A PVA loaded with cinnamon EO encapsulated in β-cyclodextrin showed a bacteriostatic effect against *S. aureus* and *E. coli* [262]. In addition, a PVA/starch film including β-cyclodextrin inclusion complex embedding lemongrass EO showed antibacterial action against *She. putrefaciens* [263]. Nano-emulsions of essential oils were used for the inclusion in active packaging. Lee et al. [264] developed hydroxypropyl methylcellulose-based films incorporating oregano EO nano-emulsions. The active film showed inhibition zones against *Sal. thyphimurium*, *E. coli*, *L. monocytogenes*, *B. cereus*, and *S. aureus*. Chitosan- *Ferulago angulata* essential oil nano-emulsion showed lower MIC and MBC values against the fish-spoilage bacteria *P. fluorescens* and *She. putrefaciens* than the corresponding coating emulsions. Moreover, the coatings, including the nano-emulsion, reduced the total viable and psychrotrophic counts of rainbow trout fillets by 3 log cfu g^−1^ after 16 days of storage at 4 °C [265]. The incorporation of *Zataria multiflora* Boiss. EO and cinnamaldehyde in the form of nano-emulsions into starch coatings reduced the growth of *L. monocytogenes*, psychrotrophic bacteria, and *Enterobacteriaceae* in chicken during cold storage [266].

## 5. Combining Effects and Hurdle Technologies

In this section, the more recent studies demonstrating the possible improvement in food safety and shelf life by combination of bioactive compounds from different plant-based extracts or by their combination with non-thermal or mild food technologies are briefly reported. 

### 5.1. Additive or Synergistic Effects

The use of combinations of antimicrobial plant extracts and their compounds showed additive or synergistic effects against spoilage and pathogenic microorganisms. This approach is cost-efficient for the food industry and adheres to the hurdle technology in inhibiting the proliferation of undesirable microorganisms, improving the preservative effects of plant antimicrobials and reducing the negative sensory effects of single plant extracts [2]. 

Additive or synergistic effects are found in combinations of plant extracts and their compounds, reducing the MIC of the plant antimicrobials. As regards the additive effects, cinnamon EO with clove EO showed an additive effect against *L. monocytogenes* [67], whereas the combination of cinnamaldehyde with 2-hydroxycinnamic acid showed additive effects against *L. monocytogenes* and *Sal. enteritidis* under in vitro conditions, but it was not effective in contaminated cooked ham [267]. Regarding the synergism among plant antimicrobials, thyme EO with cinnamon EO (0.312 g L^−1^), cinnamon EO (0.156 g L^−1^) with rosemary EO (0.625 g L^−1^) and thyme EO (0.078 g L^−1^) showed synergistic effects in inhibiting *Alt. alternata* and *P. expansum* on jujube fruit [268]. Cinnamon EO with clove EO showed synergistic antibacterial activity against *S. aureus*, *L. monocytogenes*, and *Sal. typhimurium* [269], and against *L. monocytogenes* when vanillin was combined with both EOs [67]. Synergistic effects were found using combinations of EOs compounds or isothiocyanates with phenolic acids against bacterial pathogens. The combination of thymol with gallic acid determined a synergistic effect at sub-inhibitory concentrations against *E. coli* O157:H7 and *S. aureus* on fresh-cut tomatoes [270]. Allyl isothiocyanate with o-coumaric acid showed synergism, obtaining 2 log reduction in *E. coli* O157:H7 in a dry-fermented sausage when the antimicrobial compounds were added at the concentration of 6.25 μL and 750 mg per 100 g fresh weight, respectively [271]. 

As regards the antifungal interactive effect of plant antimicrobials, a triple combination of thyme EO, cinnamon EO, and rosemary EO showed a synergistic antifungal effect against *B. cinerea* and *P. expansum*, reducing their development on pear [272]. Pinto et al. [64] demonstrated a synergistic effect in the vapour phase between thymol and γ-terpinene in binary combinations and between p-cymene, γ-terpinene, and thymol in ternary combinations against the strain *P. digitatum* ITEM 9569, which is resistant to single thyme EO exposure. The use of combinations of plant antimicrobials to control the development of spoilage and pathogenic microorganisms in foods is a research area showing rapid development. The use of combinations of plant antimicrobials can reduce the concentration of plant antimicrobials added in foods, minimizing the negative impact of these compounds on the sensory properties of foods as previously demonstrated [269,270]. Studies related to this topic are expected to increase in the future, paying attention to the effect of the combination of plant antimicrobial compounds in real food matrices, and the elucidation of the modes of action.

### 5.2. Hurdle Technologies

The “hurdle approach” in the food sector refers to the successive or simultaneous application of two or more food preservation techniques for enhancing food safety and quality using lower individual treatment intensities and for achieving multi-target, mild, and reliable preservation effects [273]. This approach was followed to reduce the dose of chemical preservatives used to control the development of spoilage microorganisms in foods [274]. Recently, several studies investigated the application of mild or non-thermal technologies in combination with the use of plant antimicrobials to control the growth of spoilage and pathogenic bacteria in food and to extend the shelf life. 

**Figure 3:**
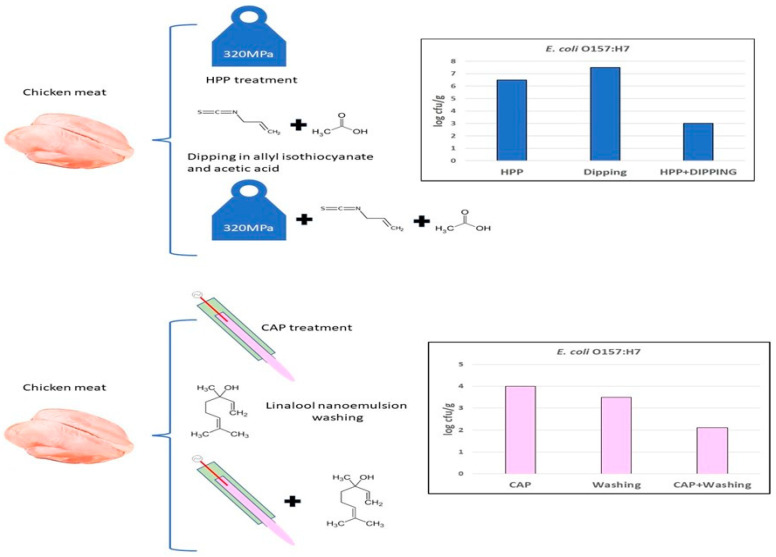
Hurdle effect of plant bioactive compounds against *E. coli* O157:H7 contaminating chicken meat when combined with high pressure or cold plasma treatments. The picture was made by drawing the main results from references [277,278].

The combined application of high-pressure homogenization (HPH) and nano-emulsions of hexanal and trans-2-hexanal inactivated *S. cerevisiae* in apple juice up to 22 days of storage, with better performance as compared to individual treatments [275]. Citral (1% *w*/*w*) combined with high-pressure processing reduced the viability of a cocktail of *E. coli* STEC in ground beef by 4–7 log cfu g^−1^, depending on the pressure level applied [276]. The treatment of ground chicken meat with 320 MPa for 23 min at 4 °C with allyl isothiocyanate and acetic acid at ca. 0.2% *w*/*w* achieved a 5-log reduction in *E. coli* O157:H7, with a better inactivation compared to single treatments [277]. As regards the application of cold plasma technology with plant antimicrobials, González-González et al. [278] found that the combined application of cold plasma and linalool nano-emulsion reduced by 3 log cfu g^−1^ the load of *E. coli* O157:H7 and *Sal. enterica* in chicken meat, while individual treatments showed limited efficacy. The effect of plant antimicrobials combined with food technologies against *E. coli* O157:H7 on chicken meat is depicted in Figure 3.

Sea bass slices packed under modified atmosphere packaging and pre-treated with cold plasma and a liposomal ethanolic coconut husk extract showed the lowest increase in *Pseudomonas* spp. and *Enterobacteriaceae* during 18 days at 4 °C in comparison to cold plasma treatment alone or the application of the liposomal ethanolic coconut husk extract [279]. On the contrary, the combined use of cinnamon EO and modified atmosphere packaging showed limited efficacy against spoilage bacteria of lean pork meat or salmon during cold storage [280]. The pulsed electric field pre-treatment of Pacific white shrimp, followed by the soaking in 1% of Chamuang (*Garcinia cowa* Roxb.) leaf extract, showed a lower increase in mesophilic, psychrophilic, *Pseudomonas* spp., *Enterobacteriaceae*, and H_2_S producing bacterial counts in comparison to individual treatments and the application of sodium metabisulfite during cold storage [281]. A synergistic effect in reducing the microbial load of *E. coli* O157:H7 was found between thyme EO nano-emulsion treatment and the sonoporation induced by ultrasounds [282]. Microwave heating at 915 MHz with carvacrol showed a synergistic effect against *E. coli* O157:H7, *Sal. typhimurium* and *L. monocytogenes* in buffered peptone water but not in hot chilli sauce [283].

Light technologies were also combined with the use of plant antimicrobials to control the contamination by foodborne pathogens. In reconstituted powdered infant formula, the load of *Cro. sakazakii* was reduced by 6.5 log cfu mL^−1^ following the combined 405 nm light-emitting diode and 9 µL mL^−1^ citral treatment for 90 min compared with untreated samples [284]. Silva-Espinosa et al. [285] found that the combined application of UV-C light and clove EO on stainless steel surface achieved a complete bacterial reduction (6.8 log cm^−2^) on biofilms of *Sal. thyphimurium*. 

The use of chemical compounds was associated with plant extracts and their compounds. The nano-emulsion of thyme EO enhanced the antimicrobial effect of slightly acidic electrolyzed water against foodborne pathogens, suggesting the formation of complexes probably through hydrophobic interactions [286]. Based on total viable counts, a shelf-life extension of 7 days was observed in fresh fish fillets treated with gaseous ozone and coated with alginate, including different EOs and citrus extract, as compared to single treatments [287].

The combination of mild or non-thermal technologies with plant antimicrobials is a recent research trend. Many of these studies showed synergistic effects in reducing the microbial load of spoilage and pathogenic microorganisms on foods. These effects can be explained by the exposure of target microorganisms to multiple hurdles and stresses. Even though more studies are necessary to understand the modes of action of these combined approaches, the enhanced antimicrobial activity of plant extracts and their compounds, when coupled with innovative technologies, allows to reduce their concentration in different foods, mitigating some of their drawbacks such as the modification of sensory characteristics. 

## 6. Regulation and Safety Issues of Plant Extracts

Plant extracts can be contaminated by different dangerous compounds, such as heavy metals, crop-protection residues, and mycotoxins. The concentration of these compounds depends on the cultivation practices employed, the geographical location of the cultivation site, the application of crop protection products, and the extraction method. In this section, data related to the contamination levels of plant extracts employed for the production of plant antimicrobials for food purposes are reported.

### 6.1. Heavy Metals and Crop-Protection Residues

Heavy metals can contaminate plant extracts. In plants that produce EOs, the uptake of metals is associated with soil contamination, and their transfer to EOs depends on the extraction technology. Moreover, the storage of EOs in metallic containers can promote the transfer of metals into the oil. As, Cd, Pb, and Hg cause toxic effects at relatively low levels. For Cd, Hg, and Pb, recommendations for safety limits in medicinal plants are imposed by European Pharmacopoeia, FAO, and the World Health Organization (WHO). Iordache et al. [25] evaluated the heavy metal content of EOs from different sites. High levels of Hg, Cr, Pb, Cu contamination were found in *Mentha × pipperita* L. EO. However, the authors concluded that the analyzed EOs could be safely consumed in the doses recommended by the manufacturers, and the content of heavy metals does not pose a significant risk to the consumer’s health. High levels of Cr, Cd, and Pb in thyme and oregano plant samples were found by Reinholds et al. [288].

Regarding the contamination levels of crop-protection residues in EOs, they depended on the agricultural practices. Conventional orange EOs contained 17 pesticides and a total concentration of 5.1 mg L^−1^, whereas organic orange EOs contained only 4 pesticides and a total concentration of 0.087 mg L^−1^ [27]. Organophosphorus and organochlorine pesticide residues were found in citrus EOs, especially those produced by cold-pressing and conventional agriculture practice, albeit with a concentration lower than 1 mg L^−1^. Tebuconazole and propiconazole co-distillated in peppermint EO with a different degree depending on the vapour pressure [289]. Cymoxanyl, dimethoate, and tebuconazole residues exceeded the maximum residue level set by the European Union in thyme samples from Poland [288]. In conventional grape skin extracts, Boscalid, Fludioxinil, Mycobutanil, and Pyraclostrobin levels exceeded the maximum residue level. However, the concentration of these crop-protection products was lower than the detection limit or the maximum residue level in organic grape skin extracts [290]. 

### 6.2. Mycotoxins

Mycotoxins can contaminate plant extracts. Different studies evaluated the mycotoxin contamination of plants such as *Mentha* sp. and *Zingiber officinale* R., which are generally used to produce EOs. Although the plant material showed levels of mycotoxins within the EU regulation limits, in some cases, the aflatoxin concentration exceeded acceptable standards [26]. In addition, the fungal contamination and mycotoxin accumulation in *Z. officinale* R. decreased the bioactive compounds of ginger [291]. Dried thyme herbs from Lebanon showed 75% of samples exceeding the limit of aflatoxin B1 for spices according to the European regulation. Similarly, the OTA level exceeded the maximum limits for Lebanese thyme and thyme mixes in 13% of the samples [292]. Zearalenone and deoxynivalenol contamination (range 10–209 µg kg^−1^) was detected in thyme samples from Poland [288]. Additional data are required to monitor the mycotoxin contamination of plant extracts (e.g., essential oils, phenolic extracts) and to establish regulatory limits.

### 6.3. Regulation

Comprehensive toxicological studies are necessary to obtain the approval of plant antimicrobials as food preservatives by the European Food Safety Authority (EFSA), the Food and Drug Administration (FDA), and the China Food Additives Association (CFAA). Many plant antimicrobials have the GRAS status for specific food applications, but their use in other food applications is not expressly approved. Indeed, plant phenolics are actually absent in the positive list of food preservatives. Regulatory authorities’ approval of plant extracts as food additives is essential to ensure consumer safety and confidence. Such authorization must be based on comprehensive safety assessments, including toxicological studies, exposure assessments, and evaluations of factors such as purity, stability, and potential allergenicity. Regulatory approval helps to guarantee that these plant extracts are safe for human consumption and meet specific quality standards. However, there can be challenges and barriers in the regulatory approval process. The complexity of plant antimicrobial mixtures and the need for extensive safety data may lead to time-consuming and costly approval processes. Global policy inequities significantly impact the approval of plant extracts as food additives. Addressing these inequities requires international cooperation, knowledge sharing, and capacity building to develop robust regulatory frameworks, protect and promote traditional knowledge, enhance research capacity in developing countries, and ensure fair and equitable access to the global market for plant-derived products. In order to improve and expedite the approval of plant extracts as food additives by authorities, several actions could be taken: encourage international collaboration and communication to harmonize regulations, establishing consistent guidelines and standards, increase investment in research and development to generate robust scientific data on plant extracts’ safety, efficacy, and potential applications, strengthen developing countries’ research and regulatory capacities through training, resources, and technical assistance, facilitate knowledge sharing and collaboration among various stakeholders, including researchers, industry professionals, and regulatory authorities, and employ advanced technologies such as data science for data-driven decision-making in the approval process. In turn, this will ensure consumer safety, promote the sustainable use of plant resources, and contribute to the growth of the global market for plant-derived products.

## 7. Conclusions and Future Directions

Plant antimicrobials gained considerable attention as promising alternatives to synthetic preservatives in the food industry, offering numerous benefits such as enhanced safety, extended shelf life, and increased consumer acceptance. The observed additive or synergistic effects between plant extracts, essential oils, and their compounds, and the successful integration of hurdle technologies, contributed to the scouting of novel and mild food preservation methods. Despite the significant progress made in the field, several research areas warrant further investigation. A deeper understanding of modes of action of plant antimicrobials and their combinations, including their effects at molecular and cellular levels on target microorganisms, is crucial for optimizing their application and enhancing their efficacy. The development of optimized formulations and delivery systems, such as nano-emulsions, encapsulation, or edible coatings, will improve stability, bioavailability, and targeted delivery of plant antimicrobials and, as a consequence, will increase their use in various food systems. A thorough examination of the impact of plant antimicrobials on the sensory properties of food is important to ensure consumer acceptance, focusing on minimizing adverse effects on taste, aroma, and texture while maintaining antimicrobial effectiveness.

Conducting comprehensive toxicological studies on plant antimicrobials, their derivatives, and combinations is essential to establish safe consumption levels and guarantee consumer safety. Additionally, addressing the challenges and barriers to regulatory approval for plant antimicrobials as food additives will accelerate their broader application in the food industry. The development of sustainable and environmentally friendly methods for extracting and producing plant antimicrobials aligns with the overall sustainability goals of the food industry. Given the knowledge on the chemical composition of plant extracts and the antimicrobial activity of plant compounds, plant antimicrobials could be recovered from agri-food by-products or from food waste, creating new value chains. Moreover, since non-edible wild plant species are often a good source of plant antimicrobial compounds, these species could be cultivated in marginal areas, promoting new cultivation practices in depressed rural territories, and also increasing the sustainable production of plant additives for the food industry. Increasing consumer awareness of plant antimicrobials’ benefits and their role in food safety and preservation will enhance market acceptance and drive demand for such products. 

The description of the results allows us to decontextualize results reported in single studies for a wider comprehension. The analysis of studies suggests that the application of plant antimicrobials, also when combined with other technologies, still needs to overcome some critical aspects. As with all reviews, this study reported a selection of case studies among many others and some of the drawbacks highlighted herein are actually under examination. 

In conclusion, we believe that this review provided useful updated information promoting a scientific evidence-based approach for researchers, aimed to understand if the use of plant antimicrobials can be scaled up from laboratory trials towards industrial applications. Further research and development efforts in these areas will help to overcome the current challenges and pave the way towards a widespread adoption of plant antimicrobials in the food industry, contributing to safer, more sustainable, and consumer-friendly foods. 

## Figures and Tables

**Table 2:** Advantages and disadvantages of the spray-drying process for producing stable and functional plant antimicrobial powders.

Plant Antimicrobial	Spray Drying Inlet Temperature (°C)	Protective Matrix	Microbial Targets	Advantages	Disadvantages	Data from Ref.
Eugenol and thymol	105	Zein/casein	*E. coli* O157:H7, *L. monocytogenes* Scott A	Good dispersion in water and good stability during storage	not reported	[223]
Eugenol	180	Whey protein/maltodextrin/chitosan	*E. coli*, *L. innocua*	High encapsulation efficiency and thermal stability	Chitosan inclusion negatively affects thermal stability, releasing and antimicrobial properties of the powder	[224]
Carvacrol	100–190	Pectin/sodium alginate	*E. coli* K12	Better thermal stability	High inlet temperature affects dissolution time and hygroscopicity	[225]
Oregano EO	100	Whey protein/maltodextrin	*E. coli*, *S. aureus*	Low residence time, high yield, low inlet temperature	Low throughput, extended processing hours, high production cost	[226]
Green tea extract	150	Maltodextrin	-	High thermal stability and reduced weight loss	not reported	[227]

## Data Availability

Not applicable.
